# The molecular chaperone ALYREF promotes R-loop resolution and maintains genome stability

**DOI:** 10.1016/j.jbc.2024.107996

**Published:** 2024-11-13

**Authors:** Jay Bhandari, Cristina Guillén-Mendoza, Kathryn Banks, Lillian Eliaz, Sierra Southwell, Darriel Eyaa, Rosa Luna, Andrés Aguilera, Xiaoyu Xue

**Affiliations:** 1 Department of Chemistry and Biochemistry, Texas State University, San Marcos, Texas, USA; 2 Centro Andaluz de Biología Molecular y Medicina Regenerativa-CABIMER, Universidad de Sevilla-CSIC-Universidad Pablo de Olavide, Seville, Spain; 3 Departamento de Genética, Facultad de Biología, Universidad de Sevilla, Seville, Spain; 4 Materials Science, Engineering, and Commercialization (MSEC) Program, Texas State University, San Marcos, Texas, USA

**Keywords:** R-loop, genome instability, ALYREF, UAP56, DNA-RNA helicase, TREX complex

## Abstract

Unscheduled R-loops usually cause DNA damage and replication stress, and are therefore a major threat to genome stability. Several RNA processing factors, including the conserved THO complex and its associated RNA and DNA-RNA helicase UAP56, prevent R-loop accumulation in cells. Here, we investigate the function of ALYREF, an RNA export adapter associated with UAP56 and the THO complex, in R-loop regulation. We demonstrate that purified ALYREF promotes UAP56-mediated R-loop dissociation *in vitro*, and this stimulation is dependent on its interaction with UAP56 and R-loops. Importantly, we show that ALYREF binds DNA-RNA hybrids and R-loops. Consistently, ALYREF depletion causes R-loop accumulation and R-loop–mediated genome instability in cells. We propose that ALYREF, apart from its known role in RNA metabolism and export, is a key cellular R-loop coregulator, which binds R-loops and stimulates UAP56-driven resolution of unscheduled R-loops during transcription.

R-loops form naturally during transcription, when nascent RNA hybridizes with the template DNA strand behind the elongating RNA polymerase II (RNAP II) (1). R-loop structures serve important physiological functions, such as class switch recombination, protection of genes from DNA methylation at GC-rich promoters, promotion of transcription termination, gene regulation, and telomere stability (2, 3, 4, 5, 6, 7, 8). However, inappropriate formation and unscheduled accumulation of R-loops perturb replication fork (RF) progression by generating R-loop–mediated RF stalling and DNA damage (9). Thus, unscheduled R-loops represent a major source of DNA damage, replication stress, and genome instability (9, 10). Importantly, the accumulation of R-loops is connected to several neurodegenerative diseases and cancer (3, 11, 12, 13, 14, 15, 16), highlighting the importance of R-loop regulation in cells.

Cells have evolved different strategies to prevent or resolve pathological R-loops. First, factors involved in RNA biogenesis or processing, including the conserved THO/transcription-complex functioning in coupling transcription and mRNA export (17, 18, 19, 20, 21), or the RNA processing factor SRSF1 (22, 23) among others (9, 24), limit the availability of the nascent RNA strand to rehybridize with the template DNA strand, thus preventing the accumulation of R-loops. Depletion of the human THO complex components results in chromosome breakage and genome instability that stem from R-loop accumulation (25, 26, 27). Second, R-loop resolvases that are of two types. One is the RNase H family enzymes, RNase H1 and RNase H2, remove R-loop structures by degrading the RNA moiety of DNA-RNA hybrids including those associated with R-loops (28, 29, 30). A large number of reports have shown that overexpression of RNase H enzymes can suppress the phenotype caused by R-loop formation in bacteria, yeast, and human cells making them an important tool for R-loop studies (*see* (31)). Recently, another class of RNases has been shown to resolve R-loops *in vitro* and *in vivo*. This is the case of DICER, which differently to any other R-loop resolvase, only acts on R-loops but not on DNA-RNA hybrids (32). The second type of R-loop resolvases is formed by specialized DNA-RNA helicases that eliminate R-loops and avoid DNA-RNA hybrid accumulation, thus alleviating the potential threat to genome integrity. These include human SETX, UAP56, FANCM, BLM, PIF1, AQR, DDX19, DHX9, DDX21, DDX23, DDX1, DDX5, DDX17, DDX18, DDX41, DDX47, and their yeast orthologs (26, 33, 34, 35, 36, 37, 38, 39, 40, 41, 42, 43, 44, 45, 46, 47, 48, 49). Some of these helicases have been shown *in vitro* to unwind DNA-RNA hybrids (26, 33, 35, 44, 45, 47, 48, 50, 51, 52, 53). Finally, and third, DNA replication–associated repair factors, such as BRCA1, BRCA2, factors in the Fanconi anemia pathway of DNA damage response, or the facilitates chromatin transcription chromatin reorganizing complex also help prevent the accumulation of R-loops and R-loop–mediated genome instability stemming from transcription-replication conflicts, in this *via* a DNA replication/repair–mediated process (38, 52, 54, 55, 56, 57).

The conserved human THO complex interacts with a number of RNA-binding proteins including a human DEAD-box RNA helicase UAP56 (Sub2 in yeast) and an essential RNA export adapter ALYREF (Yra1 in yeast) to form with RNA a larger complex termed transcription-RNA export (TREX), an intermediate structure in packaging and export of the nascent mRNA (17, 19, 58, 59). Importantly, depletion of human UAP56 leads to unscheduled R-loop accumulation and R-loop–mediated DNA damage induced by RF stalling (26). Moreover, UAP56 locates in the majority of the transcribed regions of genes from 5′ to 3′ regions, overlapping with the majority of the DNA-RNA hybrid-prone genes, indicating its essential role in preventing the cotranscriptional accumulation of unscheduled R-loops over the entire genome (26). In addition to unwinding duplex RNA structures, purified UAP56 resolves DNA-RNA hybrids as well as R-loop–mimicking structures, which are dependent on its ATPase activity (26, 60). Overexpression of WT UAP56, but not the helicase-dead mutants, suppresses R-loops and genome instability induced by depleting other RNA helicases such as DDX23, SETX, AQR, or the FANCD2 Fanconi anemia repair factor, or the messenger ribonucleoprotein (mRNP) biogenesis factor THOC1, consistent with its ability to resolve DNA-RNA hybrids and R-loops genome-wide regardless of their origin (26).

ALYREF/Yra1 is another RNA-binding protein within TREX, an intermediate mRNP particle at the TREX interface (61, 62). It is well known for its canonical function as an mRNA export adaptor that physically interacts with the conserved mRNA export receptor NXF1/Mex67 allowing the mRNA–protein complex to be translocated and exported through the nuclear pore (63, 64, 65, 66). In budding yeast, Yra1 binds DNA-RNA hybrids *in vitro* and its overexpression in yeast cells can stabilize R-loops, causing R-loop accumulation and R-loop–mediated genome instability (67). In human cells, depletion of ALYREF leads to a strong genomic instability (25), but the causes and mechanisms have not been explored. Importantly, human ALYREF interacts with UAP56 through its N and C-terminal UAP56-binding motifs (UBMs) (59, 68), and it also stimulates the ATPase and RNA-RNA unwinding activity of UAP56 (69).

Altogether, these findings suggest that ALYREF functions not only canonically to adapt mRNA export and to maintain genome stability but also whether ALYREF binds DNA-RNA hybrids or promotes R-loop resolution activity of UAP56 are unknown. Here, we systematically analyzed the role of human ALYREF in R-loop homeostasis and R-loop–mediated genome maintenance. Although purified ALYREF alone does not dissociate R-loops, we show that it stimulates UAP56-mediated R-loop resolution, which depends on the ability of ALYREF to interact with UAP56 and R-loops. We further show that ALYREF binds to DNA-RNA hybrids and R-loops with a strong affinity *in vitro*. Consistent with our *in vitro* data, cellular results show that depletion of ALYREF in human cells leads to a significant accumulation of R-loops and R-loop–mediated genome instability. Our study shows that ALYREF is an important R-loop–regulating factor required to dissociate R-loops by stimulating cotranscriptional UAP56-mediated R-loop resolution and, thus, to maintain genome stability.

## Results

### ALYREF stimulates UAP56-mediated R-loop resolution *in vitro*

**Figure 1:**
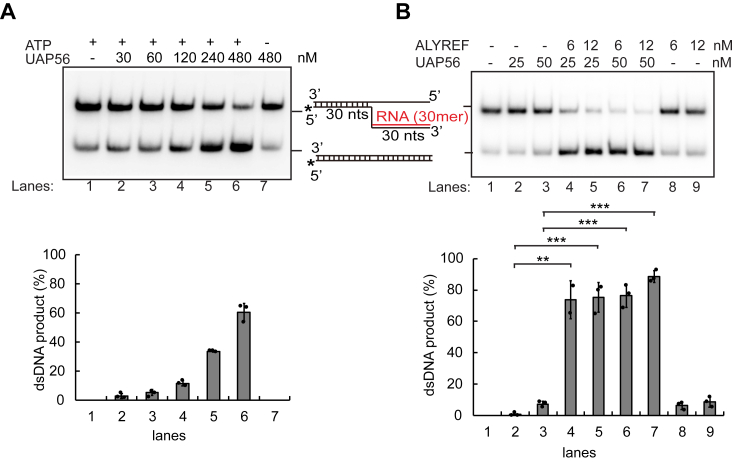
**ALYREF promotes the R-loop dissociation activity of UAP56.***A*, the R-loop dissociation activity was characterized with a serial dilution of UAP56 protein using DNA-RNA flap structures mimicking R loops as substrates. The positions of the R-loop substrates and unwound products (duplex DNA) are indicated at the *right*, where the stars show the position of the radiolabel. Gels were dried and subject to phosphorimaging analysis. The *bottom graph* shows the percentage of dsDNA product recovered after the reaction respect to the UAP56 concentration-dependent manner. *B*, ALYREF significantly promotes the R-loop dissociation activity of UAP56. ALYREF (6 or 12 nM) was incubated with UAP56 (25 or 50 nM, lanes 4–7) and used in the R-loop dissociation assay. ALYREF alone (lanes 8–9) or UAP56 alone (lanes 2–3) were tested for R-loop dissociation as control. Other details are in *A*. The percentage of dsDNA product recovered after the reaction were quantified as the mean values ± SD (n = 3 technical replicates). ∗*p* < 0.05; ∗∗*p* < 0.01; and ∗∗∗*p* < 0.001 (two-tailed paired *t* test).

ALYREF interacts directly and highly specifically with UAP56 within the TREX complex (59, 68, 70), an structure that seems to represent a large transcription intermediate containing RNA (61). Since UAP56 was previously identified as a major cotranscriptional DNA-RNA helicase that resolves R-loops genome-wide (26), we wonder whether ALYREF has any effect on UAP56-mediated R-loop resolution. UAP56 was able to dissociate a 5′ DNA-RNA flap structure that resembles a branch migratable R-loop, to yield a dsDNA product, and this is dependent on ATP hydrolysis (Fig. 1*A*) and as previously reported (26). Importantly, ALYREF and UAP56 could synergize in R-loop dissociation (Fig. 1*B*). UAP56 helicase at 25 nM could hardly dissociate the R-loop structure (5 nM) at 30 °C for 20 min; however, addition of 6 or 12 nM of ALYREF in the background of 25 nM UAP56 in the reaction could greatly enhance the R-loop dissociation, causing more than 70% of R-loop substrate turning into dsDNA products (Fig. 1*B*). It is noteworthy that 6 or 12 nM of ALYREF alone had a ∼6% to 10% of R-loop substrate background dissociation, probably resulting from the instability of the substrate and the ssRNA-binding activity of ALYREF (Fig. 1*B*). Altogether, the data indicate that UAP56 alone does not achieve its major R-loop resolvase activity, and ALYREF could strongly promote its R-loop dissociation activity.

### ALYREF binds to DNA-RNA hybrids and R-loops *in vitro*

**Figure 2:**
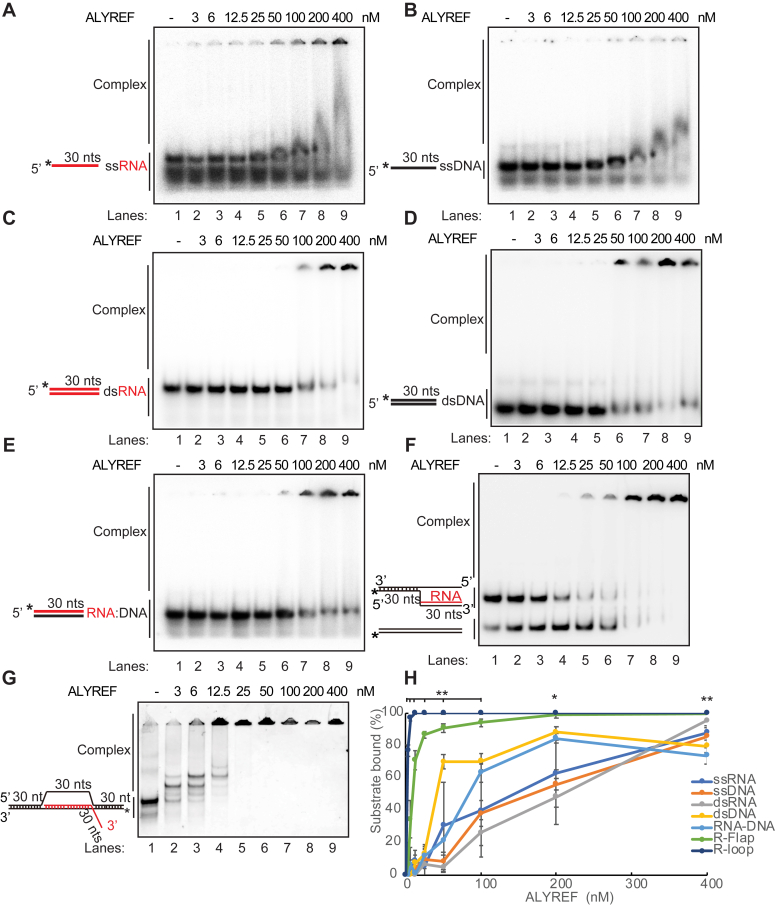
**ALYREF binds to DNA-RNA hybrids and R-loops *in vitro*.** Increasing amounts of ALYREF was incubated with 5 nM of ssRNA (*A*), ssDNA (*B*), dsRNA (*C*), dsDNA (*D*), DNA-RNA hybrids (*E*), R-Flap (*F*) or R-loop (*G*) structures, and the reaction mixtures were resolved in 7% polyacrylamide gels at 4 °C, and pictures of representative gels are shown. *H*, the percentage of the bound substrates was calculated as the percentage of intensity reduction of free DNA (or RNA) structure and was shown as the mean values ± SD (n = 3 technical replicates). Two-tailed paired *t* test between the percentage of R-loop and dsDNA binding was performed. ∗*p* < 0.05; ∗∗*p* < 0.01; and ∗∗∗*p* < 0.001.

The budding yeast ortholog of ALYREF, Yra1, was found to localize to DNA-RNA hybrid-enriched regions in cell and to have DNA-RNA hybrid binding affinity *in vitro* (67). To learn the mechanism of human ALYREF on R-loop resolution, we asked whether ALYREF could bind to DNA-RNA hybrids and R-loops *in vitro*. To test this idea, we performed an electrophoretic mobility shift assay (EMSA) to assess the nucleic acid binding affinity of ALYREF (Fig. 2, *A*–*G*). WT ALYREF was expressed in *Escherichia coli* and purified to greater than 90% homogeneity for EMSA analysis (see later). Increasing amounts of purified ALYREF were incubated with a DNA-RNA hybrid that was formed by annealing a radiolabeled RNA oligonucleotide with a complementary DNA strand (Fig. 2*E*). In parallel, we assayed ALYREF binding to the same radiolabeled RNA (ssRNA, Fig. 2*A*) or DNA strand (ssDNA, Fig. 2*B*), or to a dsRNA constructed by annealing the same radiolabeled RNA and a complementary RNA (dsRNA, Fig. 2*C*), or to a dsDNA constructed similarly (dsDNA, Fig. 2*D*). We also assayed ALYREF binding to a branch migratable R-Flap structure (Fig. 2*F*) and a nonmigratable R-loop substrate (Fig. 2*G*) structure. We observed a shift in the migration of ssRNA, ssDNA, dsRNA, dsDNA, and more importantly, DNA-RNA hybrids, R-Flap, or R-loop structures (Fig. 2*G*).

We then assessed the nucleic acid binding affinity of ALYREF, where ∼40% of ssRNA or ssDNA, or 25% of dsRNA, or 70% of dsDNA, or 63% of DNA-RNA hybrids (5 nM each) was bound by 100 nM ALYREF (Fig. 2*H*). In particular, more than 90% of the R-loop structures were bound at 6 nM of ALYREF (Fig. 2, *G* and *H*), representing an at least 16-fold increase in affinity compared to ssDNA/dsDNA or ssRNA/dsRNA. Altogether, these data indicate that ALYREF has a strong affinity to R-loop structures *in vitro*, further suggesting its important role in R-loop homeostasis.

### ALYREF interacts with UAP56 *via* its N- and C-terminal UAP56-binding domains

**Figure 3:**
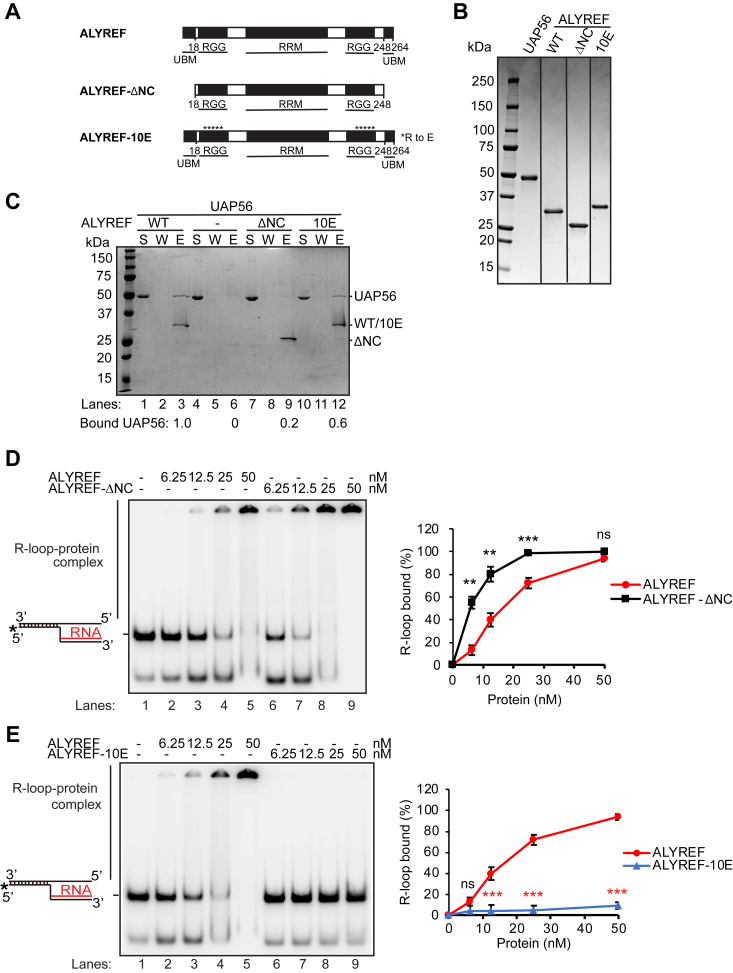
**Isolation of ALYREF mutants impaired for UAP56 interaction or nucleic acid binding.***A*, domain organization of ALYREF. The N- and C-terminal UAP56-binding motifs (UBMs), the two RGG motifs (RGG), and the RNA recognition motif (RRM) are indicated for ALYREF. The constructs of ALYREF-ΔNC, which depletes both the N- and C-terminal UBMs, and ALYREF-10E, which mutates ten conserved arginine (R) to glutamic acids (E), are shown. *B*, SDS-PAGE analysis of purified ALYREF and its mutants ALYREF-ΔNC and ALYREF-10E. *C*, Ni-NTA pull-down assay showing that ALYREF-ΔNC is defective in interaction with UAP56, whereas ALYREF-10E is only slightly affected in this regard. Purified His_6_-tagged ALYREF, ALYREF-ΔNC, or ALYREF-10E proteins bound to Ni-NTA beads were examined for their abilities to pull down UAP56. Proteins were examined by SDS-PAGE, and pictures of representative gels after Coomassie blue stain are shown. The relative ratio of bound UAP56 in the eluate (*E*) is indicated. *D* and *E*, electrophoretic mobility shift assay showing that ALYREF-ΔNC was proficient to bind R-loop structure (*D*), but ALYREF-10Ε was defective for R-loop binding (*E*). Increased amounts of WT ALYREF or ALYREF mutants (ALYREF-ΔNC or ALYREF-10Ε) were incubated with DNA-RNA flap structure mimicking R loops (5 nM) for protein–R-loop complex formation. The percentage of bound R-loops was quantified as the mean values ± SD (n = 3 technical replicates). ∗*p* < 0.05; ∗∗*p* < 0.01; and ∗∗∗*p* < 0.001 (two-tailed paired *t* test). Black stars denote significant increases, whereas red stars denote significant decreases. S, supernatant; UBM, UAP56-binding motif; W, wash.

To learn the mechanism of ALYREF on the UAP56-mediated R-loop resolution, we endeavored to generate ALYREF mutants that are differentially inactivated for either nucleic acid binding or UAP56 interaction. Previous studies had found that ALYREF contains two UAP56-binding motifs (UBMs) at its N and C terminus (68). Between the two UBMs are two conserved RGG motifs that contain multiple RGG/RG repeats (Fig. 3*A*). These multiple RGG/RG repeats are generally required for high-affinity RNA binding (71). An RNA recognition motif (RRM) is located between the two RGG motifs (Fig. 3*A*). Here, we constructed an ALYREF-ΔNC mutant with the N- and C-terminal UBMs deleted (Fig. 3*A*). The ALYREF-ΔNC mutant was expressed, purified (Fig. 3*B*), and tested for UAP56 interaction in the *in vitro* pull-down assay (Fig. 3*C*). Compared to the WT protein, the ALYREF-ΔNC mutant was largely impaired for UAP56 interaction (Fig. 3*C*). It lost about 80% of its ability to interact with UAP56.

Next, we wanted to know if ALYREF-ΔNC mutant affects its nucleic acids binding. The WT and ALYREF-ΔNC mutant were compared side by side for their interaction with R-loop structures in EMSA assays (Fig. 3*D*). The results showed that ALYREF-ΔNC has no defect, but even higher ability to interact with R-loops. About 40% of the R-Flap structures (mimicking R-loops) were bound at 12.5 nM of WT ALYREF, while 80% of the R-Flap was bound at the same 12.5 nM of ALYREF-ΔNC (Fig. 3*D*). Moreover, ALYREF-ΔNC retained the ability to interact with other DNA, RNA or DNA-RNA hybrid, and R-loop substrates (Fig. S1). Altogether, the data indicate that ALYREF-ΔNC was defective for UAP56 interaction with no defect on its nucleic acid binding activity. In fact, ALYREF-ΔNC showed significantly increased ability to bind R-loops (Fig. 3*D*). It is possibly that N- and C-terminal ends of ALYREF may impede R-loop binding, and the removal of these regions facilitates the access of R-loops to ALYREF.

### Mutations in RGG domain eliminates the DNA- and RNA-binding activity of ALYREF

ALYREF contains two conserved RGG motifs with multiple RGG/RG repeats that are generally required for high-affinity RNA binding. Between the two RGG motifs is an RRM that also can have RNA-binding activity (68). Initially, we have constructed an ALYREF-ΔRRM with the entire RRM deleted (residues 114–187, Fig. S2). Compared to the WT protein, the ALYREF-ΔRRM mutant retained its R-loop binding activity (Fig. S2*C*).

We then tested if mutation of the conserved arginine residues in the RGG domains affected the interaction with UAP56 and the nucleic acid binding activity of ALYREF. Ten arginine residues (R27, R30, R34, R36, R38, R224, R227, R231, R233, and R235) were mutated to glutamic acid (E) to generate an ALYREF-10E mutant (Fig. 3, *A* and *B*). ALYREF-10E showed a minimum defect in its ability to interact with UAP56 (Fig. 3*C*). Importantly, the EMSA assay results showed that ALYREF-10E was largely defective to bind R-Flap structure mimicking R-loop, compared to the WT ALYREF (Fig. 3*E*). Specifically, almost 94% of R-loops were bound by 50 nM WT ALYREF, while only ∼9% of R-loops were bond by 50 nM ALYREF-10E, suggesting at least 10-fold decrease in its affinity to R-loop structures (Fig. 3*E*). Furthermore, ALYREF-10E also exhibited a significant decrease of its DNA- and RNA-binding activity regarding other DNA, RNA, DNA-RNA hybrid or R-loop substrates, compared to WT ALYREF (Figs. 2, S3, S4).

### Stimulation of R-loop resolution by ALYREF requires its interaction with UAP56 and R-loops

**Figure 4:**
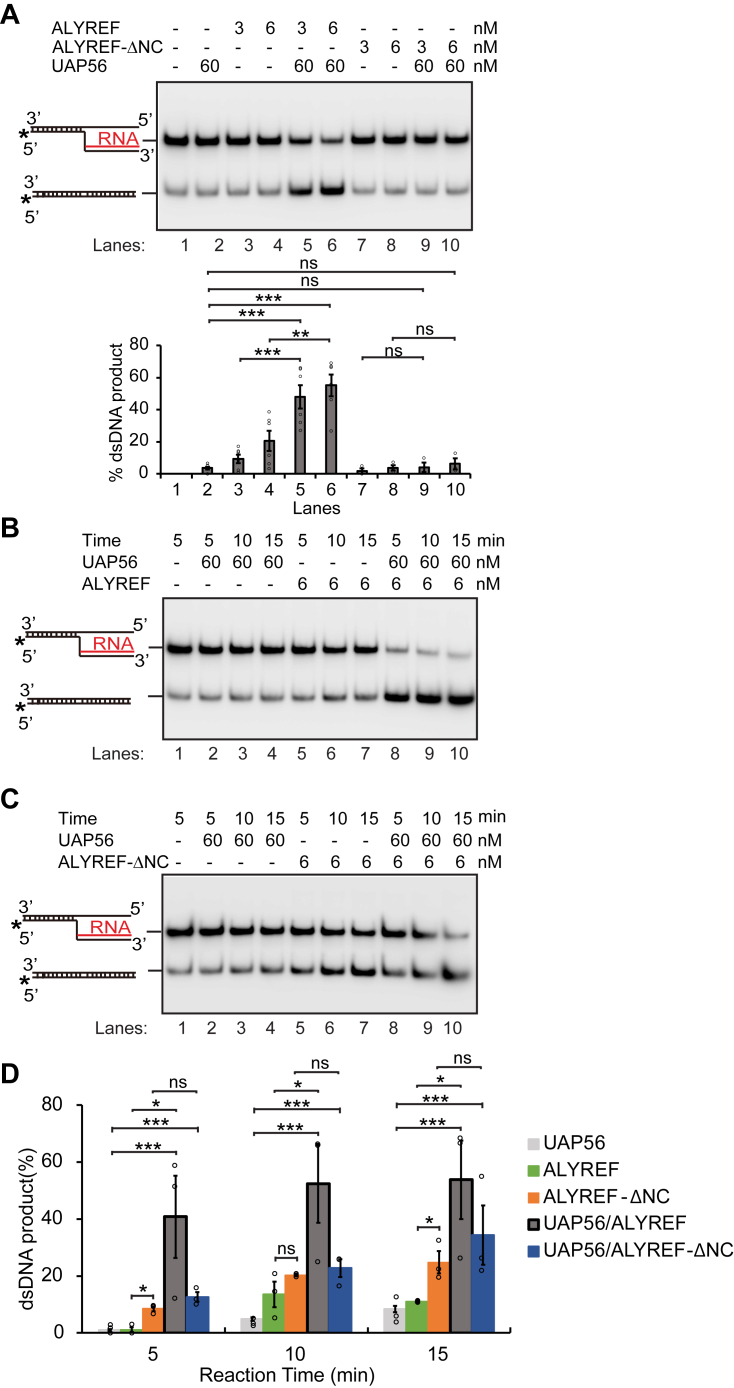
**The promotion of UAP56-mediated R-loop resolution by ALYREF requires its interaction with UAP56.***A*, ALYREF, but not its UAP56 interaction defective mutant ALYREF-ΔNC, was able to stimulate the UAP56-mediated R-loop dissociation. ALYREF or ALYREF-ΔNC (3 or 6 nM) were incubated with UAP56 (60 nM, lanes 5–6, 9–10) and used in the R-loop dissociation assay. UAP56 alone (60 nM, lane 2), ALYREF alone (3–6 nM, lanes 3–4), or ALYREF-ΔNC alone (3–6 nM, lanes 7–8) were tested for R-loop dissociation as control. The percentage of dsDNA product recovered after the reaction were quantified as the mean values ± SD (n = 3 technical replicates). ∗*p* < 0.05; ∗∗*p* < 0.01; and ∗∗∗*p* < 0.001 (two-tailed paired *t* test). *B* and *C*, a time course of R-loop dissociation assay showing that ALYREF, but not ALYREF-ΔNC, stimulated the UAP56-mediated R-loop dissociation. The R-loop dissociation were compared by UAP56 (60 nM) alone, ALYREF (6 nM) or ALYREF-ΔNC (6 nM) alone, UAP56 (60 nM) and ALYREF (6 nM) together, and UAP56 (60 nM) and ALYREF-ΔNC (6 nM) together, at 5, 10, and 15 min. The pictures of representative gels are shown. *D*, graphical representation shows the mean of the percentage of dsDNA product recovered after the reaction ± SD from at least three independent experiments from *B* and *C* (n = 3 technical replicates). ∗*p* < 0.05; ∗∗*p* < 0.01; and ∗∗∗*p* < 0.001 (two-tailed paired *t* test).

Next, we tested whether the stimulation effect of UAP56-mediated R-loop resolution by ALYREF requires its interaction with UAP56. First, we compared the R-loop dissociation by incubating (i) UAP56; (ii) UAP56 and WT ALYREF; (iii) UAP56 and ALYREF-ΔNC with same amount of 5′ DNA-RNA flap structure that mimics the R-loop structure at 30 °C for 20 min (Fig. 4*A*). As expected, we found a 3- to 5-fold increase for the R-loop dissociation by addition of WT ALYREF to the UAP56-mediated reaction (Fig. 4*A*, compare lanes 2, 3, 4 with lanes 5–6). However, ALYREF-ΔNC could not stimulate the UAP56-mediated R-loop dissociation (Fig. 4*A*, compare lane 2 with lanes 9–10), indicating the stimulation effect by ALYREF is dependent on its interaction with UAP56. Moreover, ALYREF or ALYREF-ΔNC alone had a minimum background R-loop dissociation (Fig. 4*A*, lanes 3–4, 7–8), which could be due to thermal fluctuation of the R-loop substrate and the ssRNA-binding activity of ALYREF. Importantly, the dissociation of 5′ DNA-RNA flap mediated by UAP56 and ALYREF yielded a duplex DNA product (Fig. S5). In a separate time-course experiment, WT ALYREF, but not ALYREF-ΔNC, was able to promote the UAP56-mediated R-loop dissociation (Fig. 4, *B*–*D*). It is noteworthy that at the earlier time point of 5 min, ALYREF had a 40-fold increase for the UAP56-mediated R-loop dissociation compared to a 12-fold increase by ALYREF-ΔNC (Fig. 4*D*). These results suggest that ALYREF promote the R-loop dissociation activity of UAP56 *via* its physical interaction with UAP56 by the N- and C-terminal UBMs on ALYREF. It is possible that such interactions will cause conformational changes or oligomerization of UAP56, leading it in favor of R-loop dissociation (59).

**Figure 5:**
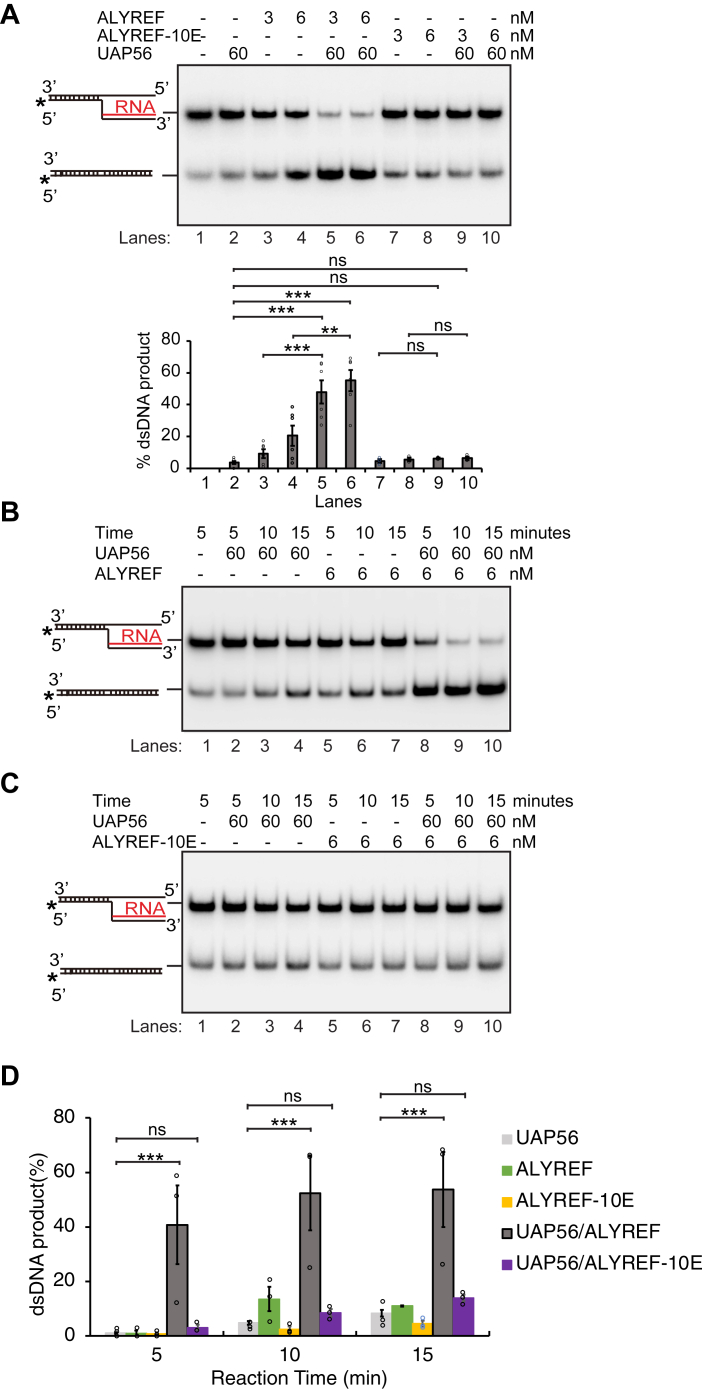
**The promotion of UAP56-mediated R-loop resolution by ALYREF requires its interaction with R-loops and nucleic acids.***A*, ALYREF, but not its nucleic acids interaction defective mutant ALYREF-10E, was able to stimulate the UAP56-mediated R-loop dissociation. ALYREF or ALYREF-10E (3 or 6 nM) were incubated with UAP56 (60 nM, lanes 5–6, 9–10) and used in the R-loop dissociation assay. UAP56 alone (60 nM, lane 2), ALYREF alone (3–6 nM, lanes 3–4), or ALYREF-10E alone (3–6 nM, lanes 7–8) were tested for R-loop dissociation as control. The percentage of dsDNA product recovered after the reaction were quantified as the mean values ± SD (n = 3 technical replicates). ∗*p* < 0.05; ∗∗*p* < 0.01; ∗∗∗*p* < 0.001 (two-tailed paired *t* test). *B* and *C*, a time course of R-loop dissociation assay showing that ALYREF, but not ALYREF-10E, stimulated the UAP56-mediated R-loop dissociation. The R-loop dissociation were compared by UAP56 (60 nM) alone, ALYREF (6 nM) or ALYREF-10E (6 nM) alone, UAP56 (60 nM) and ALYREF (6 nM) together, and UAP56 (60 nM) and ALYREF-10E (6 nM) together, at 5, 10, and 15 min. The pictures of representative gels are shown. *D*, graphical representation shows the mean of the percentage of dsDNA product recovered after the reaction ± SD from at least three independent experiments from *B* and *C* (n = 3 technical replicates). ∗*p* < 0.05; ∗∗*p* < 0.01; and ∗∗∗*p* < 0.001 (two-tailed paired *t* test).

We then examined whether the R-loop binding activity of ALYREF is required for the stimulation of UAP56-mediated R-loop dissociation. Again, the level of R-loop dissociation was compared side by side for reactions mediated by (i) UAP56, (ii) UAP56 with WT ALYREF, and (iii) UAP56 with ALYREF-10E, the mutant defective for nucleic acids binding (Fig. 5*A*). Only WT ALYREF, but not ALYREF-10E, was able to stimulate the UAP56-mediated R-loop dissociation (Fig. 5*A*, compare lane 2 with lanes 5–6 and 9–10), indicating the stimulation effect by ALYREF is dependent on its interaction with R-loops. Furthermore, ALYREF-10E alone did not have any background R-loop dissociation, comparing to WT ALYREF or ALYREF-ΔNC (Figs. 4*A* and 5*A*), suggesting that the nucleic acid binding activity of ALYREF was required for the background R-loop dissociation by ALYREF. The time-course experiment also showed that ALYREF-10E did not have any effect on UAP56-mediated R-loop dissociation (Fig. 5*D*). Altogether, these data suggest that ALYREF promotes the R-loop dissociation activity of UAP56 *via* its physical interaction with nucleic acids by the two RGG domains next to the two UBMs on ALYREF. We hypothesize that in cells ALYREF may recruit UAP56 to R-loop structures through its RGG and UBM domains, facilitating the dissociation of harmful R-loop structures and maintaining the genome stability.

### ALYREF prevents R-loop accumulation and R-loop–mediated genome instability

To learn the functional importance of the *in vitro* stimulation of UAP56-mediated R-loop resolution by ALYREF, we then tested the R-loop levels and R-loop–mediated DNA damage in ALYREF knockdown human cells. Provided the known increase in γH2AX, a marker of DNA damage, in human cells (25), we then carried out fluorescence-activated cell sorting analyses in U2OS cells depleted of ALYREF by siRNA (Fig. S6*A*) to determine whether DNA damage was related to an alteration in cell cycle progression. No significant difference was observed among the percentage of cells in G1, S or G2, neither in the intensity of 5′-ethynyl-2-deoxyuridine (EdU) labeling of siALY compared to siC cells (Fig. S6*B*), indicating that cell cycle progression is not impaired when ALYREF protein levels are reduced.

**Figure 6:**
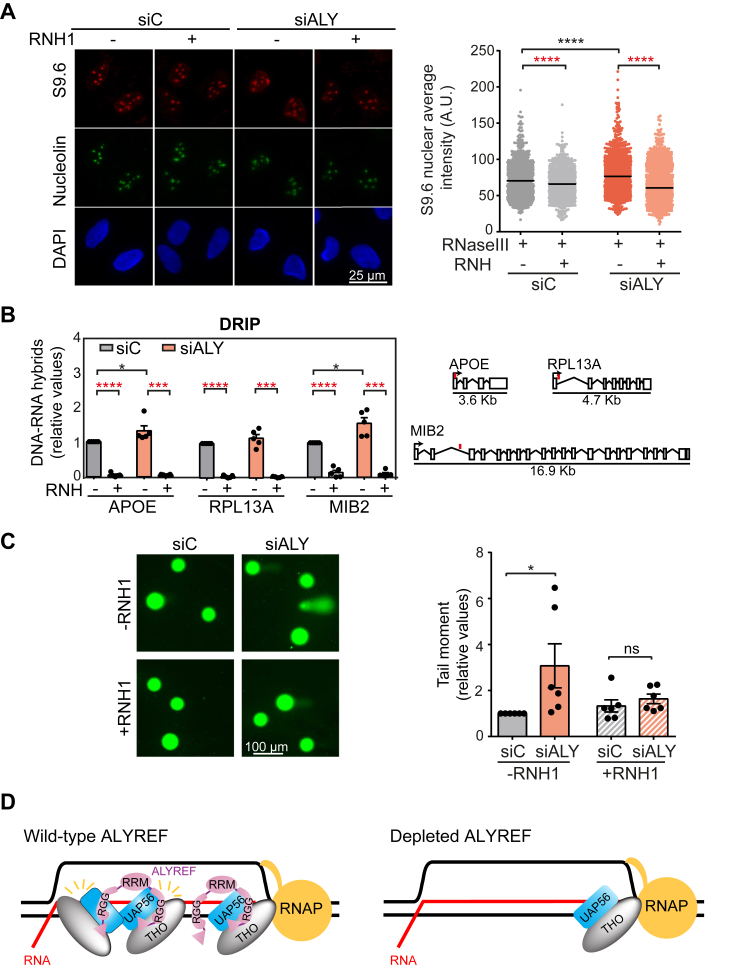
**ALYREF prevents R-loop accumulation and R-loop–mediated genome instability.***A*, representative images of immunostaining with S9.6 (*red*) and anti-nucleolin (*green*) antibodies in U2OS cells upon ALYREF knockdown. The median of S9.6 signal intensity per nucleus after nucleolar signal removal in siRNAs U2OS transfected cells and *in vitro* treated with RNase III and with or without RNase H is shown. Data from more than 200 total cells from three independent experiments is shown. ∗∗∗∗*p* < 0.0001 (Mann–Whitney *U* test, two-tailed). *Black stars* denote significant increases, whereas *red stars* denote significant decreases. *B*, relative DRIP-qPCR signal values in siALY U2OS cells at the indicated regions is shown. Samples were treated *in vitro* with RNase H prior immunoprecipitation where indicated. Signal values were normalized with respect to the siC control cells in each experiment and the mean ± SEM is plotted (n = 5). ∗*p* < 0.05 and ∗∗*p* < 0.01 (one-tailed paired *t* test). Schematic diagrams of APOE, RPL13A, and MIB2 genes are shown. Exons are depicted as *closed boxes*, the *arrows* indicate the start of transcription, and in *red* are indicated the regions where the primer pairs used for amplification were located. *C*, representative images of single-cell alkaline gel electrophoresis (comet assay) of U2OS cells upon ALYREF depletion with and without RNase H1 overexpression. Values were normalized with respect to the siC control cells in each experiment. Data are plotted as mean of the medians ± SEM (n = 6). More than 80 cells per condition were analyzed in each experiment. ∗*p* < 0.05 (one-tailed paired *t* test). *D*, model to explain the role of ALYREF in R-loop resolution during transcription. When ALYREF is present in normal amount in cells, it would bind to the R-loop structures through its two RGG domains, stimulating the interaction of UAP56 to cotranscriptionally formed R-loops through the two N- and C- terminal UBMs of ALYREF. UAP56 could be orientated or oligomerized by ALYREF in an active conformation for R-loop dissociation, which ensures that unscheduled R-loops are resolved cotranscriptionally to maintain genome stability (*left*). Instead, in siALY cells, UAP56 could not efficiently dissociate unscheduled R-loops without ALYREF, causing R-loop accumulation and R-loop–mediated genome instability (*right*). DRIP, DNA-RNA immunoprecipitation; qPCR, quantitative PCR; UBM, UAP56-binding motif.

Next, given the physical interaction of ALY with THO and UAP56, both factors involved in R-loop metabolism (25, 26), we asked whether transient depletion of ALYREF caused an R-loop increase, for which we performed immunofluorescence (IF) in U2OS cells using the S9.6 mAb that recognizes DNA-RNA hybrids. Cells were also immunostained with the anti-nucleolin antibody to identify the nucleolar signal that allowed us to specifically measure the nucleoplasm signal. A significant increase in the S9.6 nuclear signal was observed in siALY cells in comparison with siC control cells that were reduced by *in vitro* RNase III and RNase H treatments, consistent with the presence of DNA-RNA hybrids (Fig. 6*A*). To conclude this, we determined R-loop levels by the most reliable DNA-RNA immunoprecipitation (DRIP)-quantitative PCR (qPCR) method in a set of genes (*APOE*, *RPL13A*, and *MIB2*) that have been previously validated for hybrid detection (26, 72). DNA-RNA hybrids increased in siALY cells up to a significant 1.2- and 1.5-fold above the siC control levels in *APOE* and *MIB2* genes, respectively (Fig. 6*B*). Importantly, the increased DNA-RNA hybrids signals were completely reduced by *in vitro* treatment of RNase H, confirming the specificity of the assay (Fig. 6*B*). R-loop accumulation was also observed in HeLa cells depleted of ALYREF, thus validating our results in a different cell line (Fig. S6*C*). Interestingly, the overexpression of the helicase UAP56, which unwind dsRNA and DNA-RNA hybrids (26), rescued the R-loop accumulation observed in siALY cells (Fig. S6*C*), suggesting that ALYREF and UAP56 function in the same type of DNA and RNA substrates.

Next, we wondered whether the DNA-RNA hybrid accumulation caused by transient depletion of ALYREF further lead to genome instability. We assayed the levels of DNA breaks by single-cell electrophoresis (comet assay). We observed that depletion of ALYREF in U2OS cells lead to a 3-fold increase of DNA breaks in comparison with siC control cells, similar as previously reported in HeLa cells (25). Importantly, overexpression of RNase H1 suppressed the accumulation of DNA breaks indicating that the increased DNA damage was R-loop–dependent (Fig. 6*C*). This phenotype was also confirmed in HeLa cells detecting an increase in the number of үH2AX foci per cell, as previously described (25), that was reduced upon RNase H1 overexpression (Fig. S6*D*). Altogether, data supports that ALYREF depletion causes R-loop-dependent DNA damage, which is consistent with our *in vitro* data that ALYREF stimulates UAP56-mediated R-loop resolution.

## Discussion

Here, we show that ALYREF, a conserved RNA-binding protein involved in mRNA translocation and export, is an important cellular R-loop regulator that functions in maintaining cellular R-loop homeostasis. First, ALYREF is a cofactor of UAP56 promoting its ability to dissociate unscheduled R-loops (Fig. 1). Stimulation of UAP56-mediated R-loop resolution by ALYREF is dependent on its interaction with UAP56 and R-loops. This conclusion is supported by the fact that two mutants, ALYREF-ΔNC impaired of UAP56 interaction, and ALYREF-10E defective of R-loop binding, are unable to stimulate the R-loop dissociation by UAP56 *in vitro* (Figs. 4 and 5). This is further supported by the fact that depletion of ALYREF causes the accumulation of R-loops in both U2OS and HeLa cells (Figs. 6, S6). In addition, overexpression of UAP56 can completely remove the increased R-loop accumulation in ALYREF-depleted HeLa cells (Fig. S6*C*), supporting our conclusion that ALYREF prevents the accumulation of R-loops by promoting UAP56 to dissociate them (Fig. 1). Second, ALYREF has a strong affinity to DNA-RNA hybrid and R-loop structures (Fig. 2). ALYREF has been identified in different screenings for DNA-RNA hybrid–interaction proteins (34, 42, 44). It would be interesting to investigate whether ALYREF could stabilize R-loop structures in cells, as found for its yeast ortholog Yra1 (67). In summary, in siALY cells, UAP56 could not efficiently dissociate unscheduled R-loops without ALYREF, which causes the accumulation of R-loops and R-loop–mediated genome instability.

Depletion of ALYREF also causes R-loop–mediated genome instability in U2OS cells, as evidenced by the increase of DNA breaks detected by the alkaline comet assay that is reduced to almost siC levels upon RNase H1 overexpression (Fig. 6). Furthermore, the increase in the number of үH2AX foci per cell in siALY HeLa cells is also suppressed by RNase H1 (Fig. S6; (25)), a result that is not due to any indirect impact on cell cycle progression, which is unaffected in siALY and siC U2OS cells (Fig. S6). Altogether, our data suggest a role of ALYREF in preventing unscheduled R-loops and R-loop–mediated genome instability.

ALYREF interacts with UAP56 *via* its N- and C-terminal UBMs (68). Between the two UBMs is an RGG motif that contains multiple positive charged RGG/RG repeats, followed by a central RRM and a second RGG motif (Fig. 3; (68)). However, the functional importance of these domains in ALYREF has not been fully understood. Here, we have found that the two RGG domains are essential for ALYREF association with nucleic acids including R-loops, as evidenced by the fact that ALYREF-10E mutant is largely defective for nucleic acid binding (Figs. 3, S3, S4), while the RRM domain contribute less for its R-loop association (Fig. S2). It has been reported that the N- and C-terminal UBMs can separately bind UAP56 (73), raising the possibility that one ALYREF molecule may bridge two UAP56 molecules within the THO/TREX complex. This UBMs bridging model by ALYREF was first proposed according to the human core THO/TREX complex structure and the yeast Sub2-Yra1C-RNA crystal structure (59, 74) and may explain how ALYREF promotes the R-loop dissociation activity of UAP56. Importantly, our results show that the N- and C-terminal UBMs on ALYREF may impede its R-loop binding activity, as ALYREF-ΔNC showed significantly increased ability to bind R-loops (Fig. 3*D*). It is possible that removal of the N- and C-terminal UBMs facilitates the access of R-loops to ALYREF. Our results show that both UBMs and the RGG domains are indispensable for its promotion of UAP56-mediated R-loop dissociation.

Taking all together, we propose a model of how ALYREF coordinates with UAP56 for R-loop resolution that would explain the R-loop increase and genome instability of ALY-depleted cells (Fig. 6*D*). First, ALYREF’s ability to interact with UAP56 could favor the bridging of two UAP56 molecules for enhanced helicase activity. Alternatively, ALYREF could enhance UAP56 binding to R-loop structures through its two RGG domains. In this context, ALYREF may orientate UAP56 in an active conformation for a better R-loop dissociation.

UAP56 distributes all over the transcribed genes in human genome and represents a major DNA-RNA helicase that removes unscheduled R-loops formed during gene transcription (26). Our results clearly show that ALYREF could significantly stimulate the R-loop unwinding activity of UAP56 to more than 10-fold (Fig. 1), making it an important cotranscriptional R-loop regulator. Together with the THO complex, ALYREF and UAP56 may associate with each other and cooperatively remove the occasionally formed R-loops during transcription, releasing the nascent RNA from the DNA to ensure proper transcript elongation and export. In this sense, UAP56 clusters overlap with the precision nuclear run-on sequencing and RNAP II-S2P chromatin immunoprecipitation sequencing peaks that are marks of transcription elongation regions (26), and depletion of ALYREF reduces the RNAP II occupancy, and the expression of certain transcripts as determined by ChIP and run-on assays (75). Our study did not address the primary role of UAP56-THO together with ALYREF in RNA metabolism catalyzing the formation of an active and export-competent mRNP, which can also contribute to prevent the formation of R-loops by limiting the ability of the nascent RNA to hybridize back with its DNA template behind the RNAP II (9), as it may be the case for other RNA helicases of the DDX family, as recently reviewed (76). Certainly, the canonical mRNA export adaptor function of ALYREF as part of the nuclear mRNPs in cells (61, 62) and its role stimulating UAP56-mediated R-loop resolution are not mutually exclusive but actually interdependent.

The fact that UAP56 (26) and ALYREF (this study) by themselves can suppress R-loop accumulation regardless of their origin and the mode in which they are achieved in cells, suggest that they may also act independent of THO or the mRNP particle itself. Evidence suggests that THO plays a key role in recruiting UAP56-ALYREF to transcriptional sites to allow its function modulating the cotranscriptional formation of the RNA exportable mRNP particle (77). The ability of THO together with UAP56-ALYREF to promote a proper mRNP formation that would restrict the ability of the nascent RNA to hybridize back with the template DNA, together with the ability of UAP56-ALYREF to remove nonscheduled cotranscriptional DNA-RNA hybrids, support further their relevance in the cotranscriptional prevention and resolution of unscheduled R-loops by DNA-RNA unwinding to ensure genome integrity.

A question raised is why cells possess so many different DNA-RNA unwinding activities, as referred in Introduction. Provided that most such factors have dsRNA unwinding activity that fits with their main role as an RNA chaperone, we need further experimentation to define specifically the role and mechanism of action of each of them in R-loop homeostasis and R-loop–mediated genome instability, as previously discussed (9). In this context, our results strengthen the physiological relevance of THO and UAP56-ALYREF in these processes, thus providing new insights into the understanding of the mechanisms and biochemical activities by which cells remove unscheduled DNA-RNA hybrid and R-loop structures during transcription.

## Experimental procedures

### Cell cultures

This study has been performed using U2OS (ECAAC, 92022711) and HeLa (ECACC, 93021013) human cells as model systems.

Cells were cultured in Dulbecco’s modified Eagle’s medium (GIBCO) supplemented with 10% heat-inactivated fetal bovine serum (Sigma-Aldrich), 2 mM L-glutamine and 1% antibiotic-antimycotic (Biowest). Cells were grown at 37 °C and 5% CO_2_ and regularly tested for *mycoplasma* with negative results.

### Protein expression and purification

The human ALYREF-pET24b vector expressing wild type ALYREF with a C-terminal His_6_ tag was a gift from Stuart A. Wilson at the University of Sheffield. The complementary DNA of ALYREF-ΔNC depleting the coding sequence of both N-terminal 17 and the C-terminal 16 amino acids was synthesized and introduced into the pET24a vector to generate ALYREF-ΔNC-pET24a expression vector by Gene Universal. The coding sequence of ALYREF-10E harbouring 10 arginine to glutamic acid mutations (R27E, R30E, R34E, R36E, R38E, R224E, R227E, R231E, R233E, and R235E) were also synthesized and introduced into the pET24a vector to generate ALYREF-10E-pET24a expression vector. The expression vector of ALYREF-ΔRRM-pET24a–depleting residues 115 to 186 of ALYREF was constructed similarly.

The resulting ALYREF expression plasmids were introduced into *E. coli* BL21:DE3 Rosetta cells, which were grown at 37 °C to *A*_600_ = 0.8, and protein expression was induced by the addition of 0.2 mM IPTG and incubation at 16 °C for 16 h. Cells were harvested by centrifugation and all the subsequent steps were carried out at 0 to 4 °C. For lysate preparation, a cell pellet (10 g, from 2 L of cell culture) was suspended in 100 ml K buffer (20 mM KH_2_PO_4_, pH 7.4, 10% glycerol, 0.5 mM EDTA, 0.01% Igepal, 1 mM DTT) with 500 mM KCl, 1 mM PMSF and 1 tablet of Pierce Protease Inhibitor (Thermo Fisher Scientific) and then subjected to sonication (six 30 s pulses). The crude cell lysate was clarified by ultracentrifugation (100,000*g* for 45 min) and diluted with 2.5 volumes (∼250 ml) of K buffer, to reduce the KCl concentration to around 150 mM. The diluted lysate was loaded to a home packed 10 ml SP Sepharose (Cytiva) column, which was pre-equilibrated with 50 ml of K buffer plus 150 mM KCl. After washing the column with 80 ml of K buffer plus 150 mM KCl, the SP Sepharose column was applied with a linear gradient from 150 to 850 mM KCl in K buffer to elute ALYREF, and the peak fractions (from 430-540 mM KCl) were pooled and incubated with 2.5 ml of Nickel-NTA resin (Qiagen) for 1 h. The resin was washed once with 50 ml K buffer containing 500 mM KCl, once with 25 ml K buffer containing 500 mM KCl and 1 mM each of ATP and MgCl_2_, two times with 50 ml K buffer containing 500 mM KCl, once with 25 ml K buffer containing 500 mM KCl and 15 mM imidazole, before eluting the His_6_-tagged ALYREF with K buffer supplemented with 500 mM KCl and 200 mM imidazole. The eluate (12 ml) was diluted with 2.5 volumes of K buffer and loaded onto a 1 ml Mono S column (Cytiva). The Mono S column was then washed with 10 ml K buffer containing 150 mM KCl and then developed with a 25-ml gradient from 150 to 850 mM KCl in K buffer. ALYREF protein eluted at ∼550 mM KCl, and the peak fractions were pooled and concentrated to 0.5 ml and further fractionated in a 24 ml Superdex 75 column (Cytiva) in K buffer with 600 mM KCl. The ALYREF protein was eluted with a peak fraction at 10.3 ml. Fractions containing highly purified ALYREF (0.3 mg protein) were pooled, concentrated to 0.3 mg/ml, and stored in small aliquots at −80 °C. The ALYREF-ΔNC, ALYREF-10E, and ALYREF-ΔRRM mutants were purified using the same procedure with a similar yield.

Human UAP56 was purified to near homogeneity using previously described procedure (26, 60).

### DNA, RNA substrate preparation

30-mer dsRNA, 30-mer dsDNA, 30-mer RNA-DNA hybrids were prepared by annealing oligonucleotides (with one of the oligonucleotides being labeled with ^32^P) listed in Table S1. The 5′ DNA/RNA flap structure that resembles a branch migratable R-loop structure was constructed as described (38), and the three oligonucleotides (DNA1, DNA2, and R5′F) used were listed in Table S1. The 3′ R-loop structure (nonmigratable) were prepared by annealing oligonucleotides (A1′, A2′, and A4′) listed in Table S1 and purified by gel electrophoresis.

### Electrophoretic mobility shift assay

In Figures 2, S1 and S3, 3 to 400 nM ALYREF (Fig. 2), or ALYREF-ΔNC (Fig. S1), or ALYREF-10E (Fig. S3) were incubated with 5 nM of indicated DNA or RNA substrates, including 30-mer ssRNA, 30-mer ssDNA, 30-mer dsRNA, 30-mer dsDNA, 30-mer RNA-DNA hybrids, 5′ RNA-DNA flap structure that resembles a branch migratable R-loop, and nonmigratable R-loop structure (Table S1), for 10 min at 30 °C in 10 μl of buffer D (35 mM Tris–HCl at pH 7.5, 1 mM DTT, 100 μg/ml BSA, 1 mM MgCl_2_, 100 mM KCl). In Figures 3 and S2, increased amounts (as indicated) of ALYREF, or ALYREF-ΔNC, or ALYREF-10E, or ALYREF-ΔRRM were incubated with 5 nM of 5′ RNA-DNA flap structure mimicking R-loops for 10 min at 30 °C in 10 μl of buffer D. In Fig. S4, increased amounts (as indicated) of ALYREF and ALYREF-10E were incubated with 5 nM of dsRNA, dsDNA, or RNA-DNA hybrids (Table S1) for 10 min at 30 °C in 10 μl of buffer D. The reaction mixtures were resolved in 6.0% polyacrylamide gels in Tris-acetate-EDTA buffer (40 mM Tris base, 20 mM acetate acid, and 1 mM EDTA) at 4 °C. Gels were dried onto Whatman DE81 paper (Whatman International Limited) and analyzed in a Typhoon 5 Biomolecule Imager (Cytiva).

### *In vitro* R-loop resolution assay

In Figure 1, UAP56 at the indicated concentration premixed with or without ALYREF was incubated with 5 nM of the 5′ DNA/RNA flap substrate in reaction buffer R (25 mM Hepes, pH 6.5, 1 mM DTT, 2 mM ATP, 2 mM MgCl_2_, 60 mM KCl) at 30 °C for 20 min. ALYREF alone (Fig. 1*B*, lanes 8–9) was also analyzed in the reactions as control. In Figures 4*A* and 5*A*, ALYREF, or ALYREF-ΔNC, or ALYREF-10E at the indicated concentration premixed with 60 nM of UAP56 were incubated with 5 nM of the 5′ DNA/RNA flap substrate in reaction buffer R at 30 °C for 20 min. ALYREF or its mutants alone were analysed in the reactions as controls. All the above reaction mixtures were deproteinized by treatment with SDS (0.1%) and proteinase K (0.5 mg/ml) for 5 min at 30 °C and then resolved in 7% polyacrylamide gels in Tris-acetate-EDTA buffer (40 mM Tris, 20 mM Acetate acid, and 1 mM EDTA) at 4 °C. Gels were dried and subject to phosphorimaging analysis. In the time-course analysis (Figures 4, *B* and *C* and 5, *B* and *C*), UAP56 alone, ALYREF (or its ΔNC or 10E mutants) alone, or UAP56 with ALYREF (WT or its ΔNC or 10E mutants) at the indicated concentrations were incubated with 5 nM of the 5′ DNA/RNA flap substrate in reaction buffer R at 30 °C for 5, 10, and 15 min. Reaction mixtures were deproteinized before being resolved in 7% polyacrylamide gels in Tris-acetate-EDTA buffer at 4 °C and analyzed, as above.

### *In vitro* protein binding assay

For Nickel-NTA affinity pull-down assay, 2 μg of His_6_-tagged ALYREF or its variants was incubated with 4 μg of UAP56 (Fig. 3*C*) in 30 μl of K buffer (20 mM KH_2_PO_4_, pH 7.4, 10% glycerol, 0.5 mM EDTA, 0.01% Igepal, 1 mM DTT) supplemented with 200 mM KCl and 15 mM imidazole for 30 min at 4 °C. The protein mixture was incubated with 20 μl of Nickel-NTA resin (Qiagen) for 30 min at 4 °C. After washing the resin four times with 200 μl of K buffer with 200 mM KCl and 15 mM imidazole, bound proteins were eluted with 20 μl of 2% SDS. Ten percent of the supernatant (S) and eluate (E) fractions and 2% of the wash (W) fraction were analyzed by SDS-PAGE.

### siRNAs, plasmids, and transfections

Transient transfection of siRNA was performed using DharmaFECT 1 (Dharmacon) or Lipofectamine 3000 (Invitrogen) according to the manufacturer’s instructions. Lipofectamine 2000 and Lipofectamine 3000 (Invitrogen) were used for plasmid transfection. Assays were performed 72 h after siRNA transfection and 24 h after plasmid transfection.

Plasmids used are listed in Table S2. RNase H1 overexpression was driven using the plasmid pcDNA3-RNaseH1 containing the full-length RNase H1 cloned into pcDNA3 (78) or using the plasmid or pEGFP-M27-H1 (79), containing enhanced green fluorescent protein fused to RNH1, lacking the first 26 amino acids responsible for its mitochondrial localization.

### Single-cell gel electrophoresis

Alkaline single-cell gel electrophoresis or comet assays were performed as described (26). Single-cell alkaline gel electrophoresis was carried out with Comet Assay Kit (Trevigen) following manufacturer’s instructions. Images were acquired with a Leica DM6000 microscope equipped with a DFC390 camera (Leica). Comet tail moments were analyzed using Comet-score software (https://www.bioz.com/result/cometscore%20pro%20software/product/TriTek%20Corp) (version 1.0.1.60). The median of tail moment was calculated, and the mean of medians from at least three independent biological repeats is shown.

### Immunofluorescence

Cells were cultured on glass coverslips and incubated with specific antibodies [Research Resource Identifier (RRID) is detailed for each antibody]. According to the antibodies, the following methodology was applied:

S9.6 IF with RNases *in vitro* treatment requires a pre-extraction step with a pre-extraction buffer consisting of 0.5% Triton X-100, 20 mM Hepes-KOH pH 7.9, 50 mM NaCl, 3 mM MgCl_2_, 300 mM sucrose. Pre-extraction was carried out at 4 °C for 30 s or 5 min incubation in U2OS cells or HeLa, respectively. Then, cells were fixed in 2% formaldehyde for 20 min at room temperature (RT) and exchanged by ethanol 70% for 5 min at −20 °C. Coverslips were washed three times in PBS. For RNase *in vitro* treatments cells were incubated in their respective commercial buffers at 1X containing 40 U/ml RNase III (AM2290, Ambion) or 60 U/ml RNase H (M0297S, New England Biolabs), for 30 min at 37 °C cells. Coverslips were blocked in 3% BSA in PBS for at least 5 h at 4 °C and then incubated with anti-S9.6 (American Type Culture Collection Cat# Hybridoma cell Line HB-8730, RRID:AB_3095416) (1:500) and anti-nucleolin (Abcam Cat# ab50279, RRID:AB_881762) (1:1000), and when required with anti-FLAG (Abcam Cat# ab1257, RRID:AB_299216) (1:1000). Primary antibodies diluted in 3% BSA in PBS overnight at 4 °C, washed in PBS three times, and incubated with the subsequent secondary antibodies conjugated with Alexa Fluor diluted in 3% BSA in PBS (1:1000) for 1 h at RT in darkness. For S9.6 *in vitro* treatment of RNase H was used to confirm the specificity of DNA-RNA hybrid signal.

γH2AX immunostaining was performed fixing cells in 2% formaldehyde for 20 min at RT and then exchanged by ethanol 70% for 5 min at −20 °C. Coverslips were blocked in 3% BSA in PBS for 1 h. Then, cells were incubated with anti-γH2AX (BioLegend Cat# 613402, RRID:AB_315795) (1:1000) in blocking solution. When required coverslips were incubated with anti-FLAG (Sigma-Aldrich Cat# F7425, RRID:AB_439687) (1:1000) or anti-RNase H1 (Proteintech Cat# 15606-1-AP, RRID:AB_2238624) (1:500). Then, they were washed in PBS three times and incubated again with the corresponding secondary antibodies (1:1000) for 1 h at RT in darkness.

Finally, after the specific IF protocol, coverslips were washed twice with PBS 1X, stained with 4′,6-diamidino-2-phenylindole, and mounted in ProLong Gold AntiFade reagent (Invitrogen). Images were analyzed and processed with the MetaMorph v7.5.1.0 (https://es.moleculardevices.com/products/cellular-imaging-systems/high-content-analysis/metamorph-microscopy) (Molecular Probes) image analysis software.

### DNA-RNA hybrid immunoprecipitation qPCR

DRIP assays were performed by immunoprecipitating DNA-RNA hybrids using the S9.6 antibody from gently extracted and enzymatically digested DNA, treated or not with RNase H (New England Biolab) *in vitro* as described (55, 57) with minor modifications. After 72 h of siRNA transfection, pellet from a confluent 10-cm plate of cells was collected using accutase, washed in PBS, and resuspended in two eppendorfs of 800 μl of 1X Tris-EDTA. Then, 21 μl SDS 20% and 2.5 μl proteinase K (20 mg/ml) were added and pellet was incubated at 37 °C overnight. DNA was extracted gently with phenol-chloroform in phase lock tubes (VWR). Precipitated DNA was spooled on a glass rod, washed with 70% EtOH, resuspended gently in 1X Tris-EDTA at 30 °C for several hours and digested overnight at 37 °C with 50 U of HindIII, EcoRI, BsrGI, XbaI, and SspI. For the negative control, half of the DNA (16 μg) was treated with 5 μl RNase H overnight at 37 °C. In parallel, S9.6 antibody (3 μl/sample) was incubated overnight at 4 °C with Dynabeads Protein A (Invitrogen) (30 μl/sample) in 1X binding buffer. Five micrograms of the digested DNA, untreated or treated with RNase H, were bound to S9.6 antibody–dynabeads complexes during 2 h and 30 min at 4 °C for immunoprecipitation. Next, the beads were washed 3 times with 1X binding buffer. DNA was eluted in 120 μl elution buffer, treated 45 min with 7 μl proteinase K at 55 °C, and cleaned with the NucleoSpin Gel and PCR Clean-up (MACHEREY-NAGEL). One microgram of the digested DNA, untreated or treated with RNase H, was used for input DNA of each condition, in which similar proteinase K treatment and purification were performed. qPCR of immunoprecipitated (IP) DNA fragments and input DNA was performed on a 7500 Fast Real-Time PCR System (Applied Biosystems). Primers used are listed in Table S1.

10X Binding buffer: 100 mM NaPO4 pH 7.0, 1.4 M NaCl, 0.5% Triton X-100. Elution buffer: 50 mM Tris pH 8.0, 10 mM EDTA, 0.5% SDS.

DRIP quantification and normalization: Input and IP were eluted in 150 μl of 1X TE. Two microliters of Input and IP were used for qPCR. Changes in the abundance of DNA-RNA hybrids in each region were determined as the percentage of input recovered for each IP sample using the equation % input = 2[(CtINPUT – log2DF) CtIP] × 100. Ct = threshold cycle; DF = dilution factor.

### Flow cytometry assays

For cell-cycle analysis, asynchronous cells transfected with the corresponding siRNAs, as indicated in “siRNAs, plasmids, and transfections,” were incubated with EdU (10 μM) for 20 min. Then, cells were collected and fixed in 70% ethanol (−20 °C, >1 h). For the staining, cells were washed twice with PBS 1X and then blocked with 3%BSA/PBS containing 0.05% Tween-20 for 30 min at RT, and washed and labeled with Click-it EdU Imaging kit (C10337, Invitrogen) following manufacturer’s instructions. Finally, DNA was stained with 4′,6-diamidino-2-phenylindole (1 μg/ml) and 0.05% Tween-20. Cells were scored by Sorter cytometer (LSRFortessa X-20; BD).

### Western blot

Western blot were performed using standard procedures. Immobilon-FL polyvinylidene fluoride (PVDF) membranes were blocked with Oddyssey PBS blocking commercial buffer (LI-COR, Cat#927-4000) and then primary antibodies were incubated for 2 h at RT or overnight at 4 °C with anti-ALYREF (Abcam Cat# ab202894, RRID:AB_3094572) (1:4000). Anti-actin (Sigma-Aldrich Cat# A3853, RRID:AB_262137) (1:2000) were used as loading controls, and Ponceau was used to determine the loading amount. Then, membranes were washed three times with PBS 1X and incubated with secondary antibodies for 1 h in darkness (at 1:5000 whether primary antibody is ≤ 1:1000, or 1:15,000 whether primary antibody is at >1:1000). Primary and secondary antibodies were incubated in the blocking buffer with 0.1% Tween-20. To rehybridize the membrane, stripping commercial buffer (Nalgene, Cat#928-40028) was used for 8 min shacking at RT.

### Quantification and statistical analysis

For electrophoretic mobility shift assay and *in vitro* R-loop resolution assay, data are expressed as mean ± SD. Student’s *t* test two-tailed was applied for comparisons of two independent groups. The statistical test used in each experiment is mentioned in the figure legend. In general, a *p* value <0.05 was considered as statistically significant (∗∗∗*p* < 0.001; ∗∗*p* < 0.01; and ∗*p* < 0.05). Specific replicate numbers (n) for each experiment can be found in the corresponding figure legends.

For S9.6 IFs and γH2AX foci/cell analysis, statistically significant differences were assessed with nonparametric Mann–Whitney *U* test one or two-tailed and medians are shown. For DRIP assay, single-cell electrophoresis assays and fluorescence-activated cell sorting analysis, Student’s *t* test one or two-tailed was applied for comparisons of two independent groups. Statistical analysis using Student’s *t* test are represented as mean with SEM as error bars. In all of them, data were analyzed with EXCEL (Microsoft, https://www.microsoft.com/en-us/microsoft-365) or GraphPad Prism (https://www.graphpad.com) software. The statistical test used in each experiment is mentioned in the figure legend. In general, a *p* value <0.05 was considered as statistically significant (∗∗∗∗*p* < 0.0001; ∗∗∗*p* < 0.001; ∗∗*p* < 0.01; and ∗*p* < 0.05). Specific replicate numbers (n) for each experiment can be found in the corresponding figure legends.

## Data availability

The data used and/or analyzed in the current study are available from the corresponding author (xiaoyu.xue@txstate.edu) on reasonable request.

## Supporting information

This article contains supporting information (26, 78, 79).

## Conflict of interest

The authors declare that they have no conflicts of interest with the contents of this article.
