# Supplementary material for: The molecular chaperone ALYREF promotes R-loop resolution and maintains genome stability
Source: J Biol Chem. 2024 Nov 13;300(12):107996. doi: 10.1016/j.jbc.2024.107996 (PMC11665464; doi:10.1016/j.jbc.2024.107996)
Supplement: Supporting information [file mmc1.docx]

**­­­**

**Supporting Information**

**The molecular chaperone ALYREF promotes R-loop resolution and maintains genome stability**

Jay Bhandari^1,5^, Cristina Guillén-Mendoza^2,3,5^, Kathryn Banks^1,5^, Lillian Eliaz^1^, Sierra Southwell^1^, Darriel Eyaa^1^, Rosa Luna^2,3^, Andrés Aguilera^2,3^, Xiaoyu Xue^1,4,*^

**Supporting Tables**

**Table S1. Oligonucleotides used in this study**

| **Oligonucleotides** | **Source** | **Use** |
| --- | --- | --- |
| ssRNA:  5′-GGUCCCACCACCAGGUGGGCAAAGAUGUCC-3′ (RNA) | IDT | EMSA assay |
| ssDNA:  5′-GGTCCCACCACCAGGTGGGCAAAGATGTCC-3′ | IDT | EMSA assay |
| dsRNA:  5′-GGUCCCACCACCAGGUGGGCAAAGAUGUCC-3′ (RNA)  5’-GGACAUCUUUGCCCACCUGGUGGUGGGACC-3′ (RNA) | IDT | EMSA assay |
| dsDNA:  5′-GGTCCCACCACCAGGTGGGCAAAGATGTCC-3′  5′-GGACATCTTTGCCCACCTGGTGGTGGGACC-3′ | IDT | EMSA assay |
| RNA-DNA hybrid:  5′-GGUCCCACCACCAGGUGGGCAAAGAUGUCC-3′ (RNA)  5′-GGACATCTTTGCCCACCTGGTGGTGGGACC-3′ | IDT | EMSA assay |
| 5′ RNA-DNA flap:  (DNA1) 5′-ACGCTGCCGAATTCTACCAGTGCCTTGCTAGGA CATCTTTGCCCACCTGGTGGTGGGACC-3′  (DNA2) 5′- GGTCCCACCACCAGGTGGGCAAAGATGTCCCA GCAAGGCACTGGTAGAATTCGGCAGCGT-3′  (R5’F) 5′-GGUCCCACCACCAGGUGGGCAAAGAUGUCC-3′ (RNA) | IDT | EMSA assay, in vitro R-loop resolution assay |
| 3′ R-loop:  (A1′) 5′- CATTGCATATTTAAAACATGTTGGATCCCACGTTG CATGCTGATAGCCTACTAGAGCTGCATGAATTCAAATGACCTCTTATCAAGTGAC-3′  (A2′) 5′- GTCACTTGATAAGAGGTCATTTGAATTCATGGCTT AGAGCTTAATTGCTGAATCTGGTGCTGGGATCCAACATGTTTTAAATATGCAATG -3′  (A4′) 5′- GCACCAGAUUCAGCAAUUAAGCUCUAAGCCGCU GACGGCUCGAUGCUGAUCGUAGCAUCG -3′ (RNA) | IDT | EMSA assay |
| ON-TARGETplus Non-targeting Pool (D-001810-10) against target sequences:  5′- UGGUUUACAUGUCGACUAA-3′;  5′-UGGUUUACAUGUUGUGUGA-3′;  5′-UGGUUUACAUGUUUUCUGA-3′;  5′-UGGUUUACAUGUUUUCCUA-3′ | Dharmacon | siRNA depletion |
| ON-TARGETplus Human ALYREF SMART pool (L-012078-00) against target sequences:  5′-UCUCAGACGCCGAUAUUCA-3′  5′-GUUAAACAGACCAGCAAAU-3′  5′-GGAACUCUUUGCUGAAUUU-3′  5′-CAAAACAACUUCCCGACAA-3′ | Dharmacon | siRNA depletion |
| APOE Fwd  5′-GGGAGCCCTATAATTGGACAAGT-3′  APOE Rv  5′-CCCGACTGCGCTTCTCA-3′ | Condalab | DRIP |
| RPL13A Fwd  5′-GCTTCCAGCACAGGACAGGTAT-3′  RPL13A Rv  5′-CACCCACTACCCGAGTTCAAG-3′ | Condalab | DRIP |
| MIB2 Fwd  5′-CTCTCCTTGTCTGGGGCTC-3′  5′-CTGCCTCCCTCACCTGTC-3′ | Condalab | DRIP |

**Table S2. Plasmids used in this study**

| **Recombinant DNA** | **Source** |
| --- | --- |
| pCDNA3 | Invitrogen |
| pCDNA3-RNaseH1 | (1) |
| pEGFP-C1 | Clontech |
| pEGFP-M27-H1 | (2) |
| pFLAG-CMV-6A | Sigma |
| pUAP56-FLAG | (3) |
| ALYREF-His_6_-pET24b | This study |
| ALYREF-ΔNC-His_6_-pET24a | This study |
| ALYREF-10E-His_6_-pET24a | This study |
| ALYREF-ΔRRM-His_6_-pET24a | This study |
| pGEX-KG-UAP56 | This study |

**Supporting Figures**

**
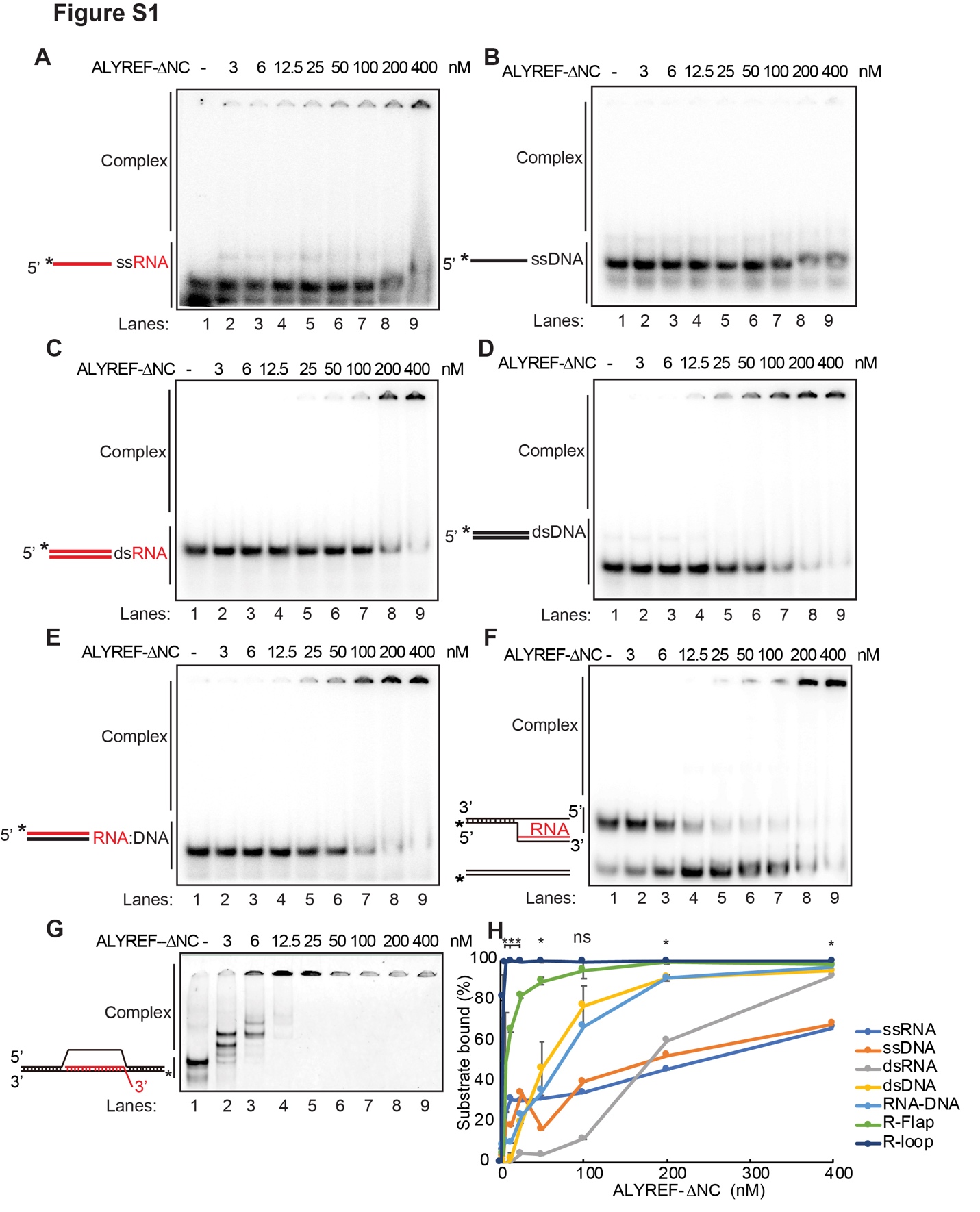
**

**Figure S1.** **ALYREF-ΔNC was proficient to bind to DNA-RNA hybrids and R-loops in vitro**. Increasing amounts of ALYREF-ΔNC was incubated with 5 nM of ssRNA **(A)**, ssDNA **(B)**, dsRNA **(C)**, dsDNA **(D)**, DNA-RNA hybrids **(E)**, R-Flap **(F)** or R-loop **(G)** structures, and the reaction mixture were resolved in 7% polyacrylamide gels at 4°C, and pictures of representative gels are shown. **(H)** The percentage of the bound substrates was calculated as the percentage of intensity reduction of free DNA (or RNA) structure, and was shown as the mean values ± SD (n = 2 technical replicates for dsDNA, R-Flap and R-loop). Two-tailed paired t-test between the percentage of R-loop and dsDNA binding was performed. ∗P < 0.05; ∗∗P < 0.01; ∗∗∗P < 0.001.

**
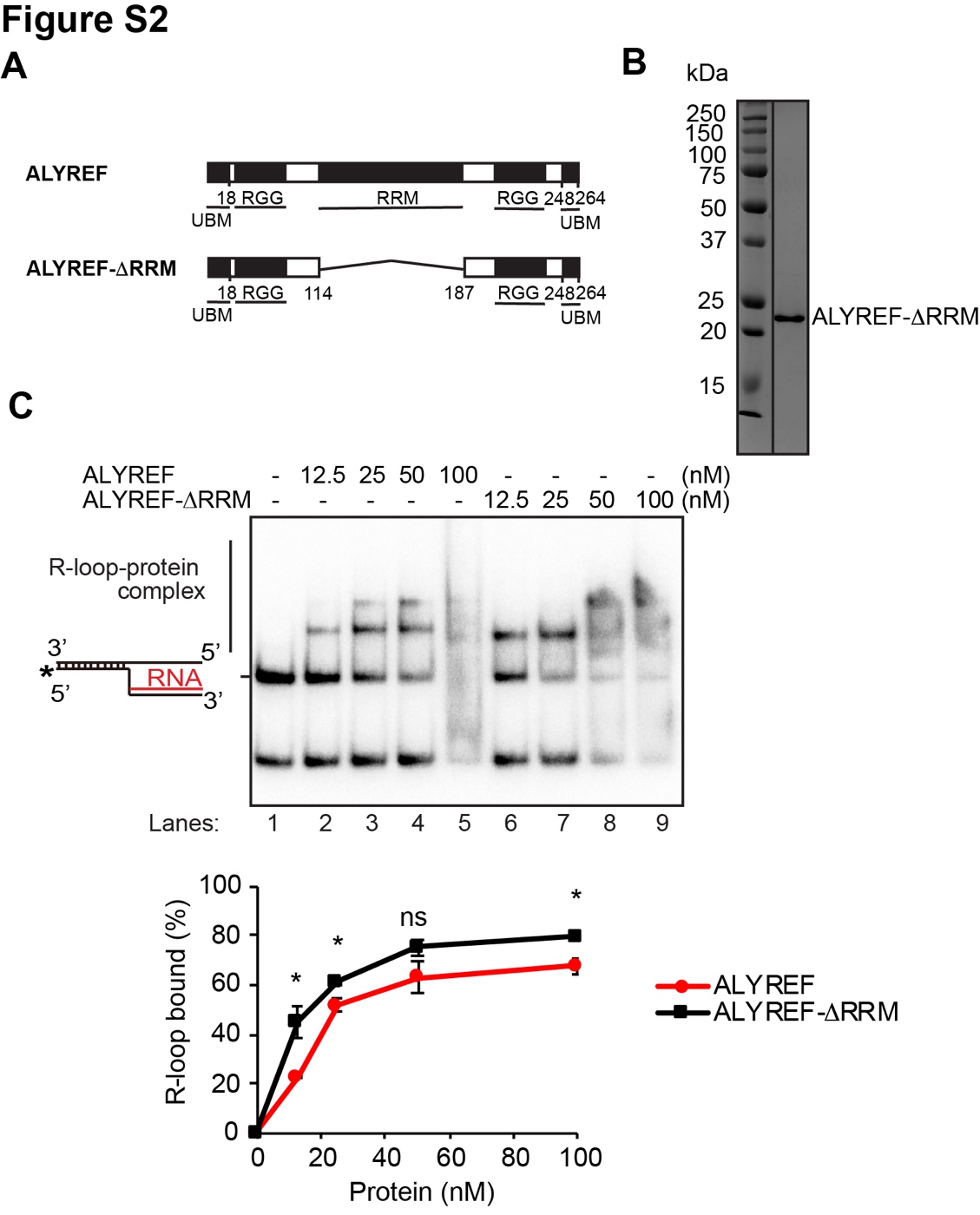
**

**Figure S2.** **ALYREF-ΔRRM depleting the entire RNA recognition motif still retains the R-loop binding activity. (A)** Domain organization of ALYREF and ALYREF-ΔRRM. ALYREF-ΔRRM depletes residues 114-187, the entire RRM. **(B)** SDS-PAGE analysis of purified ALYREF-ΔRRM. **(C)** Electrophoretic mobility-shift assay showing that ALYREF-ΔRRM was proficient to bind R-loop structure, compared to that of wild type ALYREF. The percentage of the bound substrates was quantified as the mean values ± SD (n = 3 technical replicates). ∗P < 0.05; ∗∗P < 0.01; ∗∗∗P < 0.001 (two-tailed paired t-test).

**
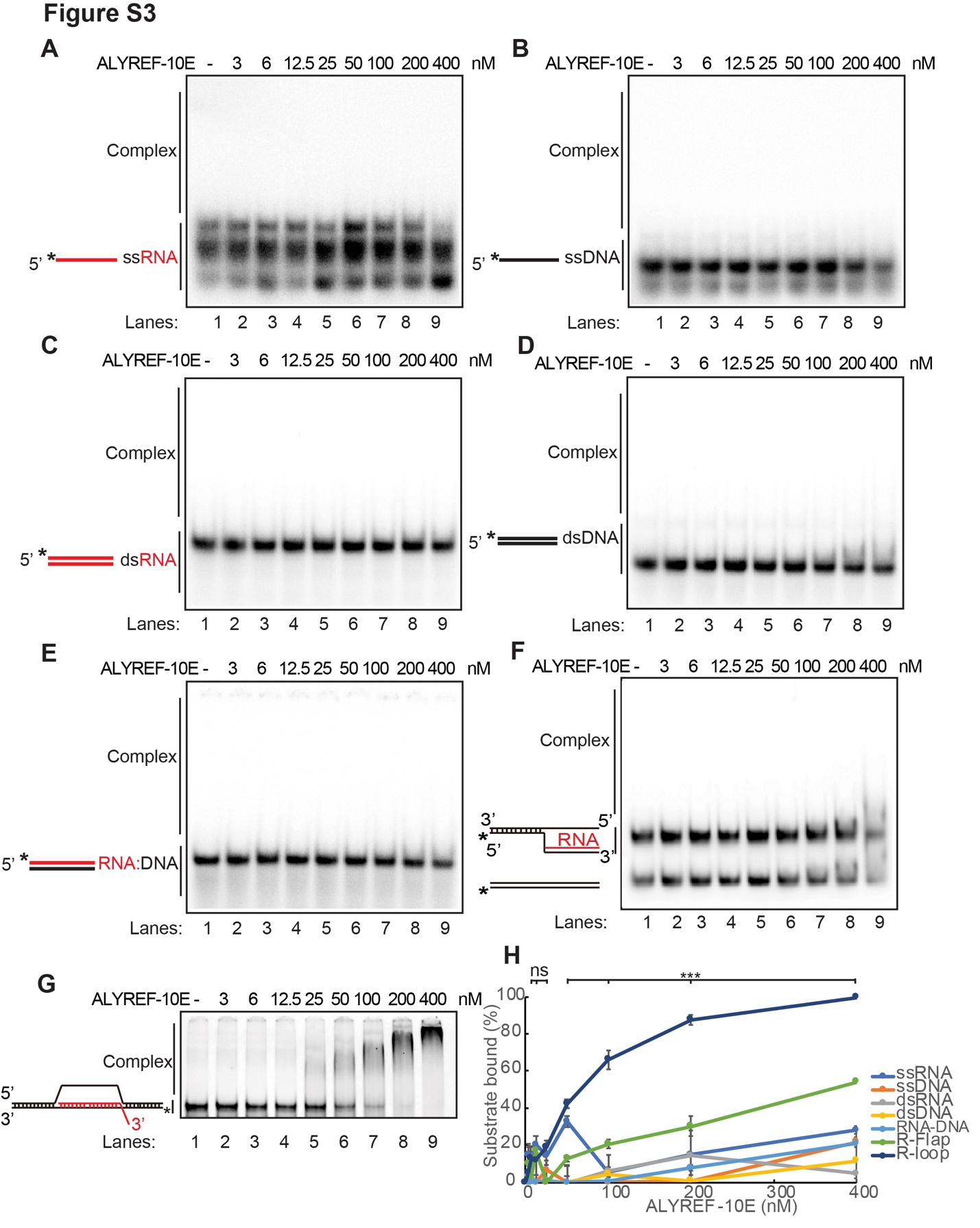
**

**Figure S3.** **ALYREF-10E was defective to bind to nucleic acids in vitro**. Increasing amounts of ALYREF-10E was incubated with 5 nM of ssRNA **(A)**, ssDNA **(B)**, dsRNA **(C)**, dsDNA **(D)**, DNA-RNA hybrids **(E)**, R-Flap **(F)** or R-loop **(G)** structures, and the reaction mixture were resolved in 7% polyacrylamide gels at 4°C, and pictures of representative gels are shown. **(H)** The percentage of the bound substrates was calculated as the percentage of intensity reduction of free DNA (or RNA) structure, and was shown as the mean values ± SD (n = 3 technical replicates). Two-tailed paired t-test between the percentage of R-loop and dsDNA binding was performed. ∗P < 0.05; ∗∗P < 0.01; ∗∗∗P < 0.001.

**
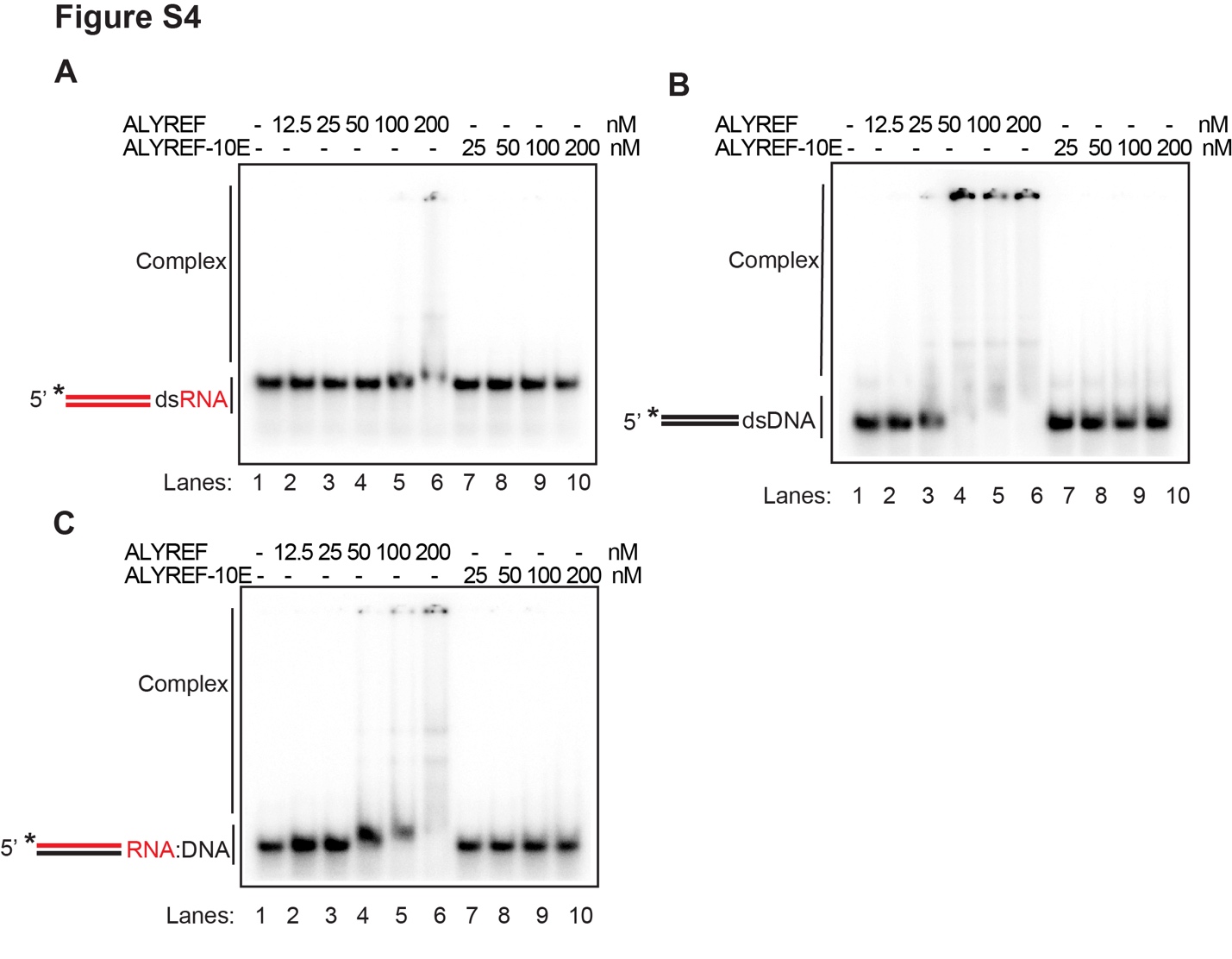
**

**Figure S4. ALYREF-10E was defective to bind to dsRNA, dsDNA and DNA-RNA hybrids compared to wild type ALYREF. (A-C)** Electrophoretic mobility-shift assay showing that ALYREF-10E was defective to bind dsRNA (A), dsDNA (B), or DNA-RNA hybrids (C). A serial dilution of wild type ALYREF or ALYREF-10E were incubated with dsRNA, or dsDNA, or DNA-RNA hybrids (5 nM) for protein-nucleic acids complex formation, and pictures of representative gels are shown.

**
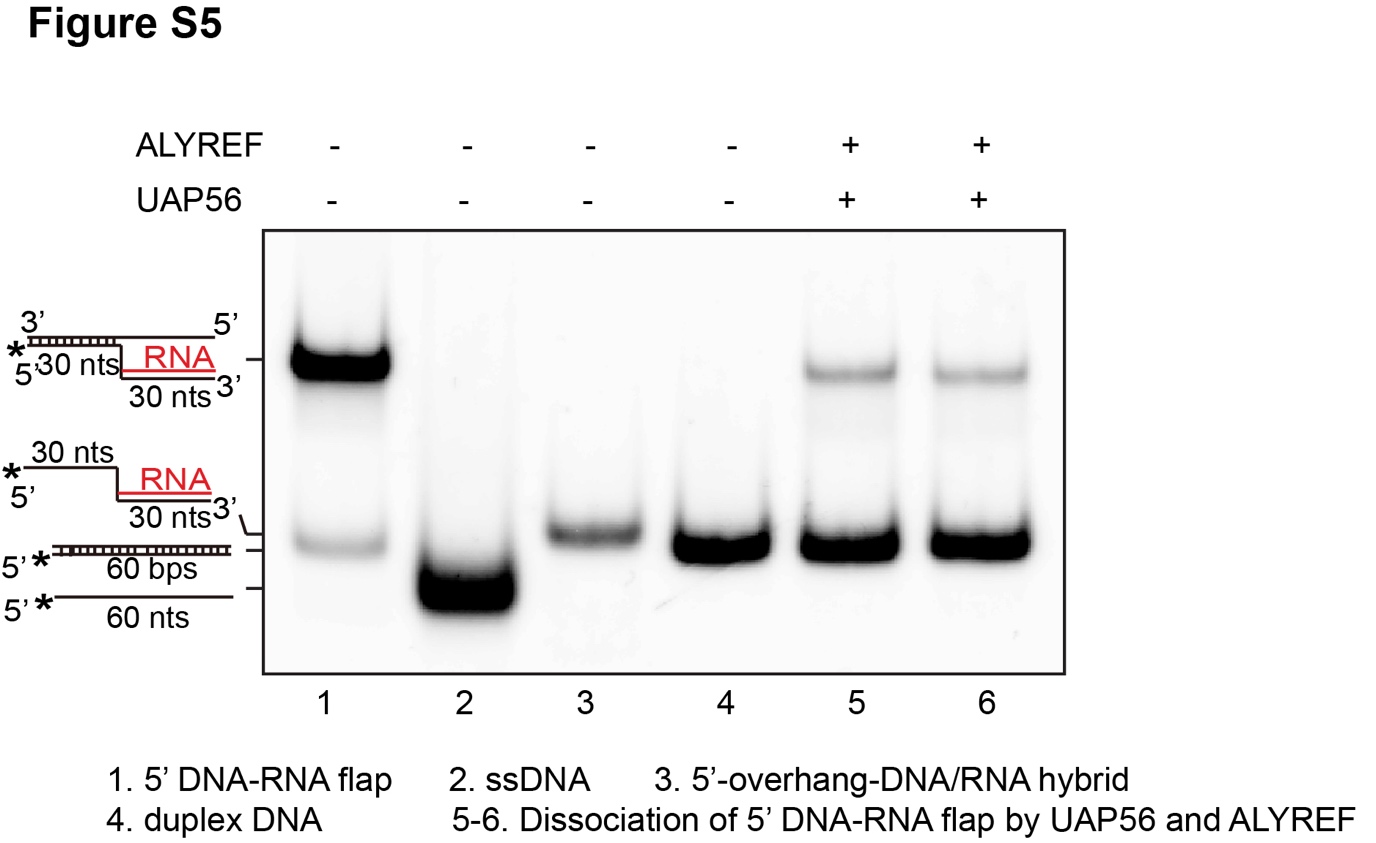
**

**Figure S5. UAP56 and ALYREF dissociates 5′ DNA-RNA flap substrate to yield a duplex DNA product.** Note that the 5′-overhang DNA/RNA hybrid was just above the duplex DNA product, while the ssDNA was below the duplex DNA. Lanes 5-6, UAP56 (60 nM) and ALYREF (6 nM) were incubated and used in the 5′ DNA-RNA flap dissociation assay. Lane 5 and lane 6 were two repeats of the same reaction.

**
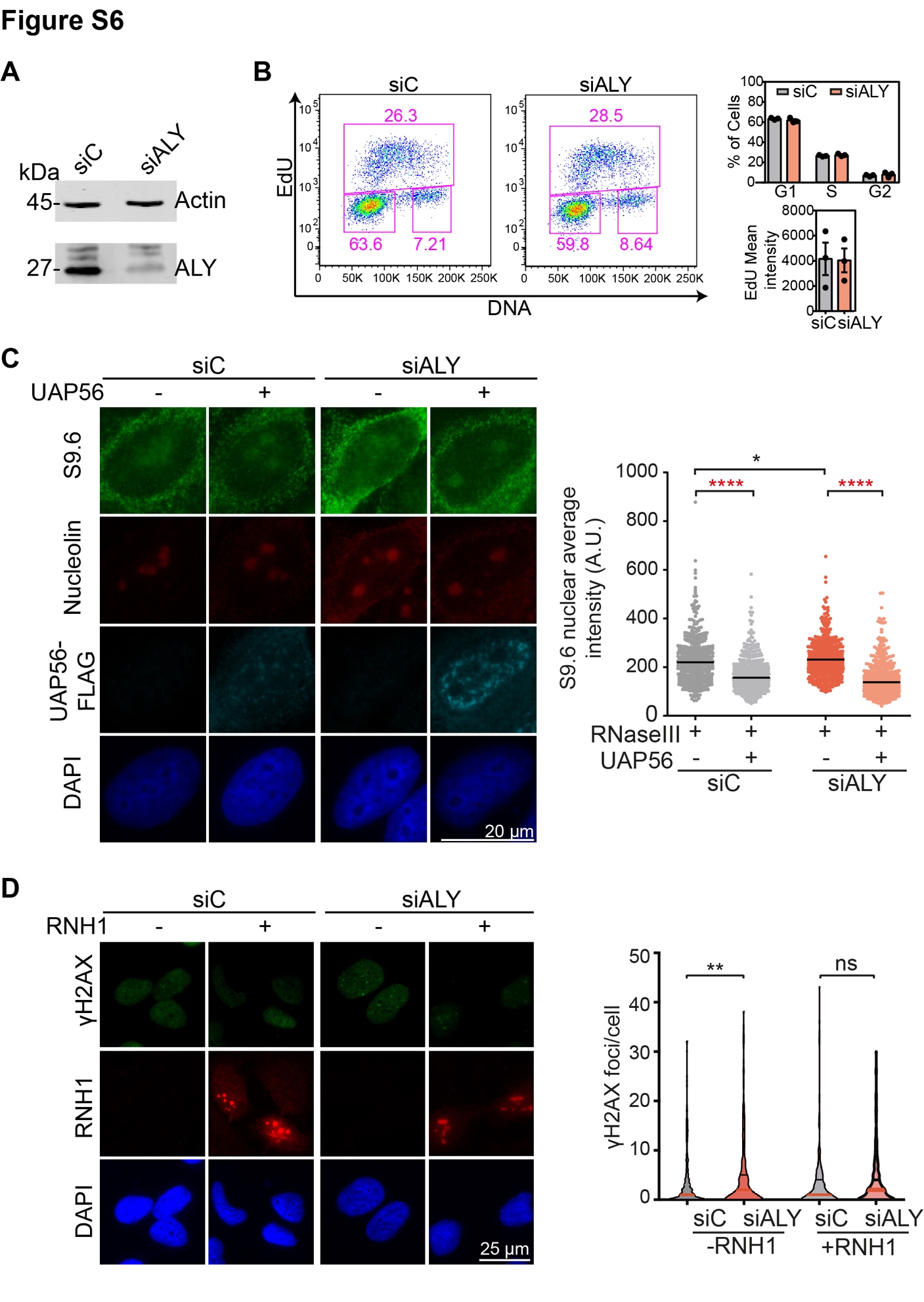
**

**Figure S6. ALYREF prevents R-loop accumulation and R-loop-mediated genome instability. (A)** Western blot analysis showing ALYREF expression levels of U2OS cells transfected with control and ALYREF siRNAs. Actin protein was used as a loading control. **(B)** FACS analysis of asynchronously growing U2OS cell cultures of control (siC) and ALYREF depleted cells (siALY) after 20 min of incubation with 20mM 5’-ethynyl-2-deoxyuridine (EdU). The percentages of cells in G1, S and G2 phases are depicted in upper panel and the EdU intensity from EdU positive cells is shown in the lower panel. Data are plotted as mean ± SEM (n=3) (two-tailed paired t-test). **(C)** Representative images of immunostaining with S9.6 (green), anti-nucleolin (red) and anti-FLAG (cyan) antibodies in HeLa cells upon ALYREF depletion. Data from 400 total cells from four independent experiments is shown. *P < 0.05; ****P < 0.0001 (Mann-Whitney U-test, one-tailed). **(D)** Representative images of immunostaining with үH2AX (green) antibody in siALY HeLa cells. Quantification of the number of үH2AX foci per cell in HeLa transfected cells with the indicated siRNAs and with or without RNase H1 overexpression is represented. Bold black lines indicate the median (n=3) **P < 0.01. The statistical significance of the difference was calculated with Mann-Whitney U-test two-tailed.

**References**

1. ten Asbroek, A. L., van Groenigen, M., Nooij, M., and Baas, F. (2002) The involvement of human ribonucleases H1 and H2 in the variation of response of cells to antisense phosphorothioate oligonucleotides. *Eur J Biochem* **269**, 583-592

2. Cerritelli, S. M., Frolova, E. G., Feng, C., Grinberg, A., Love, P. E., and Crouch, R. J. (2003) Failure to produce mitochondrial DNA results in embryonic lethality in Rnaseh1 null mice. *Mol Cell* **11**, 807-815

3. Perez-Calero, C., Bayona-Feliu, A., Xue, X., Barroso, S. I., Munoz, S., Gonzalez-Basallote, V. M., Sung, P., and Aguilera, A. (2020) UAP56/DDX39B is a major cotranscriptional RNA-DNA helicase that unwinds harmful R loops genome-wide. *Genes & development* **34**, 898-912
